# Access to primary care for socioeconomically disadvantaged older people in rural areas: a realist review

**DOI:** 10.1136/bmjopen-2015-010652

**Published:** 2016-05-17

**Authors:** John A Ford, Geoff Wong, Andy P Jones, Nick Steel

**Affiliations:** 1 Department of Public Health and Primary Care, University of East Anglia, Norwich, UK; 2 Nuffield Department of Primary Care Health Sciences, University of Oxford, Oxford, UK

**Keywords:** PRIMARY CARE

## Abstract

**Objective:**

The aim of this review is to identify and understand the contexts that effect access to high-quality primary care for socioeconomically disadvantaged older people in rural areas.

**Design:**

A realist review.

**Data sources:**

MEDLINE and EMBASE electronic databases and grey literature (from inception to December 2014).

**Eligibility criteria for selecting studies:**

Broad inclusion criteria were used to allow articles which were not specific, but might be relevant to the population of interest to be considered. Studies meeting the inclusion criteria were assessed for rigour and relevance and coded for concepts relating to context, mechanism or outcome.

**Analysis:**

An overarching patient pathway was generated and used as the basis to explore contexts, causal mechanisms and outcomes.

**Results:**

162 articles were included. Most were from the USA or the UK, cross-sectional in design and presented subgroup data by age, rurality or deprivation. From these studies, a patient pathway was generated which included 7 steps (problem identified, decision to seek help, actively seek help, obtain appointment, get to appointment, primary care interaction and outcome). Important contexts were stoicism, education status, expectations of ageing, financial resources, understanding the healthcare system, access to suitable transport, capacity within practice, the booking system and experience of healthcare. Prominent causal mechanisms were health literacy, perceived convenience, patient empowerment and responsiveness of the practice.

**Conclusions:**

Socioeconomically disadvantaged older people in rural areas face personal, community and healthcare barriers that limit their access to primary care. Initiatives should be targeted at local contextual factors to help individuals recognise problems, feel welcome, navigate the healthcare system, book appointments easily, access appropriate transport and have sufficient time with professional staff to improve their experience of healthcare; all of which will require dedicated primary care resources.

**Strengths and limitations of this study**

- A broad search was used to avoid missing major concepts.
- The programme theory generated is transferable to other settings.
- Using a realist review allowed the dynamic nature of access to healthcare to be explored.
- There was a lack of evidence specifically focusing on socioeconomically disadvantaged older people in rural areas.
- The context–mechanism–outcome configurations could not fully elucidate each complex interaction.

## Background

Improving primary care access, defined as ‘the timely use of personal health services to achieve the best possible outcome’,^1^ has become increasingly popular because of its potential to reduce hospital admissions.^2–5^ In the UK, policies to improve access have included walk-in centres, extended opening, polyclinics, 48 h access and, most recently, the Prime Minister's Challenge Fund which awards grants to local organisations to improve access to primary care (total budget £100 million).^6^ Most of these initiatives are available to the whole population but do not target groups at high risk. A review of equality of access to healthcare in the UK found that rural individuals, older people and those in lower socioeconomic groups have poorer access to healthcare.^7^ When these coexist, there is likely to be intersectionality, where complex determinants of health relate, intersect and reinforce each other,^8^ leading to delayed diagnosis,^9^ poor quality of care,^10^ higher mortality^11^ and greater inequality.^12^ National data on this group do not exist, but by triangulating data,^13^ ^14^ we estimate that there are ∼316 000 socioeconomically disadvantaged older people living in rural areas in England.

A recent systematic review assessing primary care access^15^ categorised barriers as patient factors (eg, sociodemographics), organisational factors (eg, appointment system), financial factors (eg, no health insurance), workforce factors (eg, technical skills) and geographical factors (eg, distance to services). As with other reviews,^16^ this listed the barriers, but did not encompass the dynamic, iterative and multidimensional nature of access.^17^ ^18^ This reflects the traditional systematic review methodology which aims to pool data to achieve an overall result, rather than explore and explain underlying causal processes.

A realist review seeks to explore the underlying causes for observed outcomes and when these might occur by reviewing published and grey literature.^19^ It is designed to answers questions such as ‘how?’, ‘why?’, ‘for whom?’, ‘in what circumstances?’ and ‘to what extent?’ programmes or interventions ‘work’. Through a review of the literature, an overarching programme theory is developed which is gradually refined using data drawn from documents included as the review progresses. Within this programme theory, a realist logic of analysis is used to explore outcome patterns. In brief, mechanisms cause outcomes to occur, but the relevant mechanisms will only be ‘triggered’ under the right contexts. When applying a realist logic of analysis, a factor is only assigned the conceptual label of context if there are sufficient data to support the inference that it triggers a mechanism that causes an outcome of interest (ie, one that is relevant to and found within a programme theory). The analytic building blocks are context–mechanism–outcome configurations (CMOCs).^20^ These are propositions which describe what works (or happens), for whom and in what contexts and why? Contexts are conditions that trigger or modify the behaviour of mechanisms. In this realist review, we are particularly interested in identifying and understanding the contexts that act as barriers and facilitators of access to primary care. We believe that realist methods are ideal for examining access to healthcare because they can accommodate the complex and dynamic nature of access to primary care.

We aim to use a realist review to explore the contexts that influence access to primary care for socioeconomically disadvantaged older people in rural areas by seeking to answer the following questions:

1. What are the barriers and facilitators to accessing high-quality primary care for socioeconomically disadvantaged older people in rural areas?
2. What are the underlying mechanisms, why do they occur and how do they vary in different contexts?

The purpose is to understand the process of accessing primary care, rather than how to achieve a certain outcome. We did not aim to fully elucidate every underlying mechanism, but rather take a broad overview. The review is not limited to factors which are uniquely rural, since this may overlook important issues such as ease of booking an appointment. This realist review is part of a larger research programme which includes an ongoing cohort analysis, semistructured interviews and focus groups to develop an intervention that will be tested within a feasibility study.^21^

## Methods

### Programme theory development

To develop the programme theory, an initial rough theory was first produced by JAF based on prior knowledge and an initial scoping search and subsequently discussed with GW, APJ and NS. For the scoping search, we undertook a narrow search in MEDLINE and search for reports and policy documents using an internet search engine (Google) to identify key resources and understand the breadth of literature on this topic. Documents of interest were read by JAF and discussed with the research team. Key theory, such as the Aday and Andersen Framework,^22^ informed the initial rough theory through the use of their ‘predisposing’, ‘enabling’ and ‘need’ concepts. Based on our full search, programme theory was developed using a patient pathway that logically mapped out all the steps a patient needed to go through to access care. During the review, drawing on the data in the included studies, we then gradually and iteratively refined this patient pathway into a realist programme theory that included CMOCs.

### Searching

The databases MEDLINE, MEDLINE in Process and EMBASE were searched from inception to December 2014. Search terms were initially piloted and refined to increase sensitivity. Search terms used in MEDLINE are shown in the online supplementary appendix 1. Grey literature was searched using a search engine (Google) and a targeted search of specific websites (eg, Kings Fund, Nuffield Trust and Royal College of General Practitioners). References within included documents were screened for relevance.

10.1136/bmjopen-2015-010652.supp1 Supplementary data

### Selection and appraisal of documents

All titles and abstracts were screened and articles included if they were judged to possibly contain relevant data on access to primary care in socioeconomically disadvantaged older people in rural areas. Studies did not have to include all components (ie, primary care, deprivation, older people and rural areas) because initial scoping suggested that a narrow inclusion criteria would have excluded important concepts such as ease of booking an appointment. For example, a study was eligible for inclusion if it included both rural and urban areas as long as the concepts described were relevant to socioeconomically disadvantaged older people in rural areas. Only studies published in English were included. Studies primarily focused on care homes or low-income countries were excluded. After titles and abstracts screening, we retrieved the full text of seemingly relevant articles. One author (JAF) screened all titles and abstracts. Included studies were rechecked in light of their relevance and extent to which they did actually contain data that would inform programme theory development.^20^ The purpose of screening and appraising was not to identify an exhaustive set of studies, but rather reach conceptual saturation in which sufficient evidence is identified to meet the aims of the review.^19^ After screening and rechecking, we agreed that conceptual saturation had been reached.

### Data extraction and analysis

Study characteristics were extracted into a prespecified Excel spreadsheet that was piloted before use and included publication year, country, participants' details, study design and healthcare system.

Sections of relevant text were identified from included articles and coded using QRS NVivo (NVivo qualitative data analysis Software [program]. Version 10 version: QSR International Pty Ltd, 2012). Some codes were derived inductively (originating from the included studies) whereas others were deductive (originating from the initial rough theory). Codes were refined based on emerging concepts throughout the analysis period. Coded text was chosen based on the follow questions:

Is the section of text referring to context, mechanism or outcome?
What is the CMOC (partial or complete) for it?
(A) How does this (full or partial) CMOC relate to the patient pathway? (B) Are there data which support how the CMOC relates to the patient pathway? (C) In light of this CMOC and any supporting data, does the patient pathway need to be changed?
(A) Is the evidence sufficiently trustworthy and rigorous to change the CMOC? (B) Is the evidence sufficiently trustworthy and rigorous to justify changing the patient pathway?

An overarching patient pathway was developed from the data using the NVIVO coded text and the analysis aimed to find data to corroborate, refute or refine the patient pathway into a realist programme theory by gradually and iteratively building CMOCs for each step in the patient pathway. To generate the CMOCs for each step, we started with the outcome and worked backwards. Data and sections of text from the extraction phase were interpreted as relating to context, mechanism or outcome. Most sections of text described the context–outcome process without exploring the underlying mechanism, and in these situations, we sought relevant data from other included studies to identify mechanisms. We then made inferences as to what the complete CMOC might be for each step. For example, if data were interpreted as relating to context, then the next analytic task was to assess which outcome the context was related to and what the mechanism might be. Any substantive or formal theory identified during the search was used to assist in programme theory development if relevant. Included studies were re-examined throughout the analysis and programme theory refinement period using an iterative, cyclical process to seek out data to enable judgements to be made about the relevance (contributes to the research questions), rigour (the data used in programme theory development had been generated using methods that were credible and trustworthy) and importance of emerging concepts. In other words, the analysis continually asked whether there were data to warrant modifying a CMOC and/or the programme theory.

The CMOCs were discussed with the research team, which included patient representatives, and these fed into the iterative, cyclical process of searching, data extraction, analysis and programme theory development. Patient representatives were recruited from Older People's Forums in Norfolk and contributed to the design and interpretation of the research. Findings are reported in accordance with the RAMESES publication standards.^23^ Ethics approval was not required for this study.

## Results

### Search results and study characteristics

**Figure 1:**
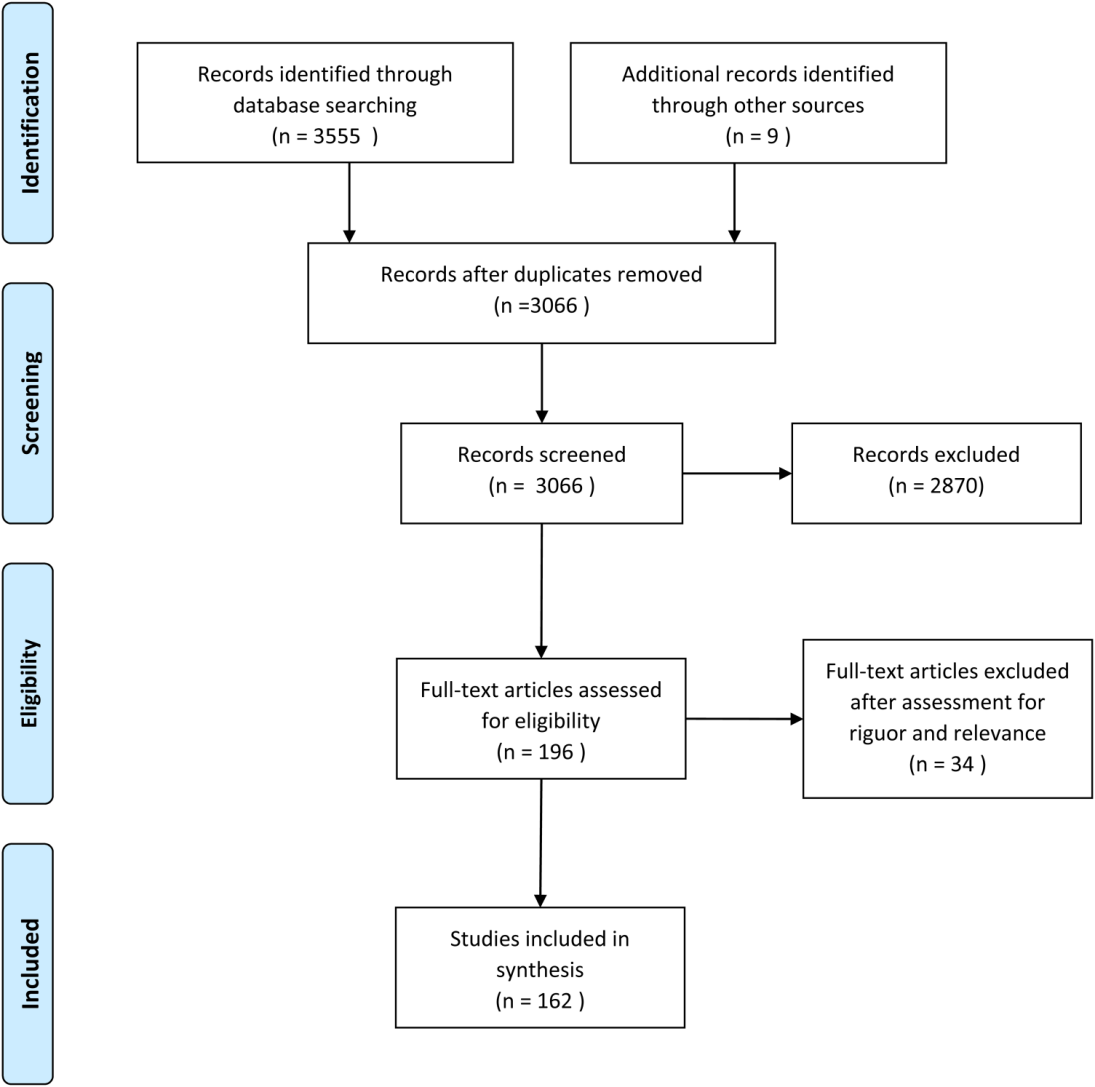
PRISMA diagram.

In total, 3065 titles and abstracts were screened (figure 1) leading to full-text review of 196 articles. Thirty-four articles were excluded after assessment for relevance and rigour leaving 162 to be included. Most studies were from the USA or the UK, cross-sectional in design, not disease-specific and provided subgroups of older adults, socioeconomic disadvantaged people, rurality or primary care (table 1). No studies were found that only included socioeconomically disadvantaged older people in rural areas accessing primary care.

**Table 1:** Study characteristics

	Number of studies
*Country*	
USA	49
UK	48
Canada	19
Australia	9
New Zealand	9
Other	28
*Study type*	
Cross-sectional	85
Analysis of routine data	24
Qualitative	22
Cohort	16
Editorial or discussion paper	3
Other	12
*Health problem*	
Any health problem	114
Urgent health problems	10
Ambulatory care sensitive conditions	8
Mental health	5
COPD	3
Diabetes	3
Heart disease	3
Other	16
*Age*	
All adults	111
Older adults only	51
*Socioeconomic position*	
All adults	150
Socioeconomically disadvantaged only	12
*Rurality*	
Rural and urban	137
Rural only	13
Urban only	12
*Gender*	
Both	157
Female only	4
Male only	1
*Health domain*	
Primary care only	69
Primary and secondary	93
*Subgroup analysis of relevant population*	
Yes	114
No	48
Total	162

COPD, chronic obstructive pulmonary disease.

### From patient pathway to realist programme theory

**Figure 2:**
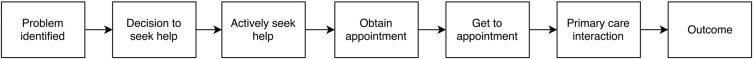
Patient pathway.

Thirty-four articles provided data that were synthesised and used to create the patient pathway (figure 2) from which the realist programme theory would be iteratively developed. The final step named ‘outcome’ refers to the result of a primary care interaction such as treatment, referral, reassurance or dissatisfaction. The first three steps (problem identified, decision to seek help and actively seek help) were described in Broadhurst^24^ and used by Kovandzic *et al*^25^ in a study exploring access to mental health services for hard to reach groups. The remaining steps were mainly developed from key sources.^5^ ^26–29^ For example, Buetow *et al*^27^ summarised previous literature evaluating access to primary care as falling into three categories (1) organisation processes, such as appointment systems (obtaining an appointment); (2) geographical literature around physical access (getting to the appointment) and (3) social and cultural influences (cutting across both obtaining an appointment and getting to it). These data contributed to the ‘obtain appointment’ and ‘get to appointment’ steps.

This patient pathway is transferable to most primary care populations and the concepts described below are particularly relevant to socioeconomically disadvantaged older people in rural areas. The patient pathway is shown as a linear pathway for simplicity, but it is clear that access to primary care is considerably more complex and dynamic.^26^ ^30^ For example, a patient with an intermittent problem (such as chest pain) may transit between the first three steps (problem identified, decision to seek help and actively seek help) for days or weeks as they decide if the problem is real, warrants assessment and what the most appropriate service is.

### Context–mechanism–outcome configurations

**Figure 3:**
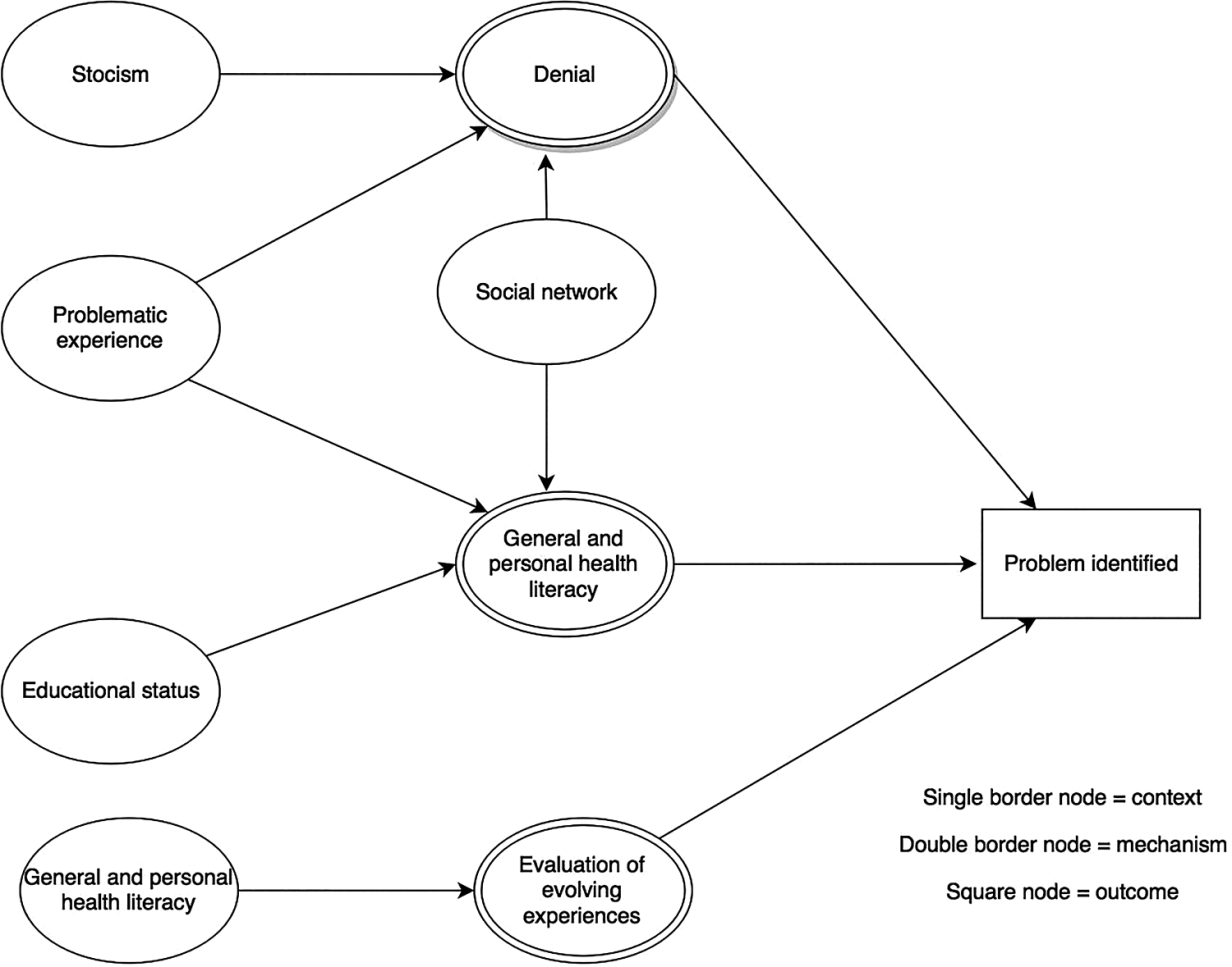
Context–mechanism–outcome configuration for problem identified.

**Figure 4:**
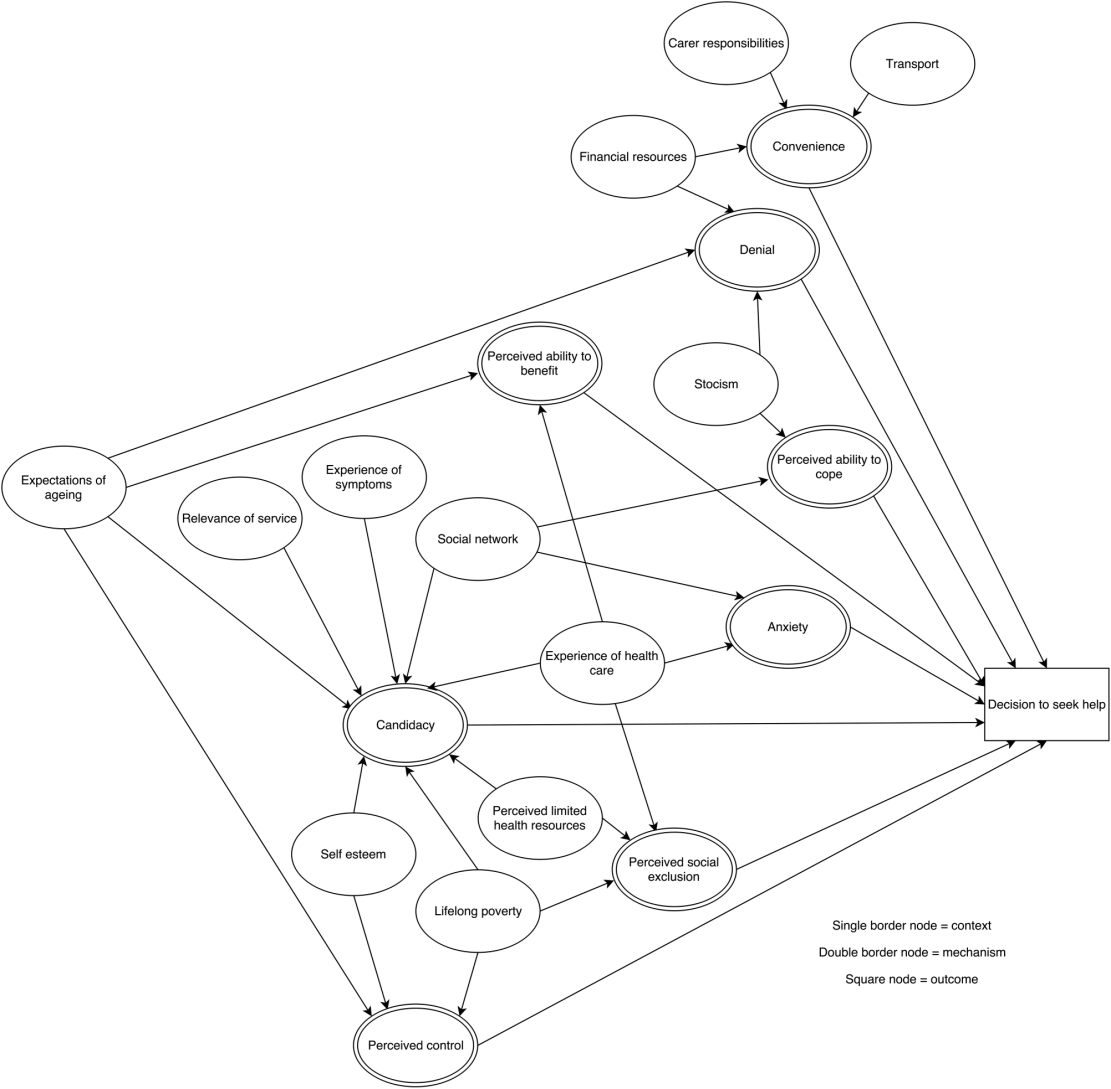
Context–mechanism–outcome configuration for decision to seek help.

**Figure 5:**
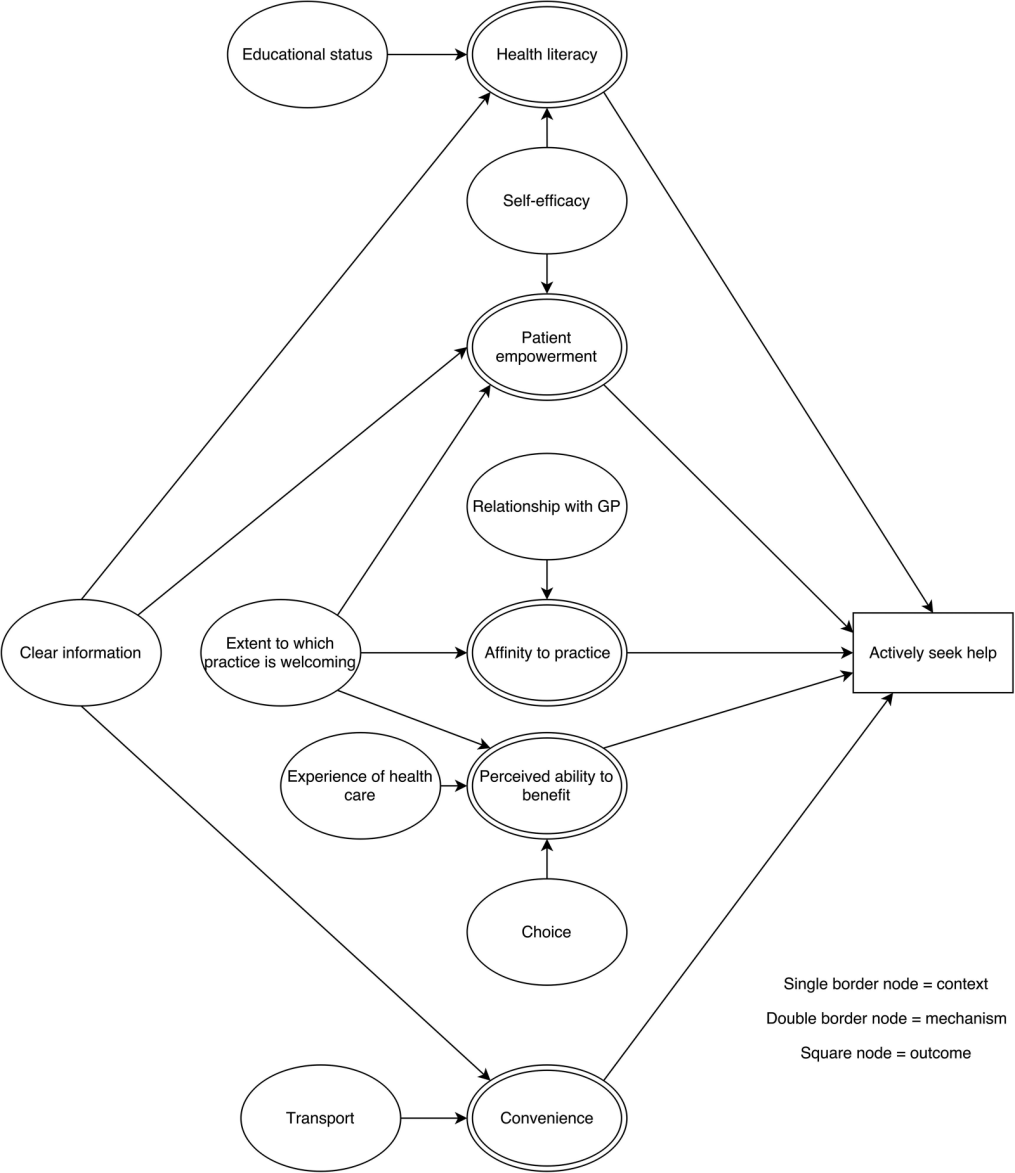
Context–mechanism–outcome configuration for actively seek help. GP, general practitioner.

**Figure 6:**
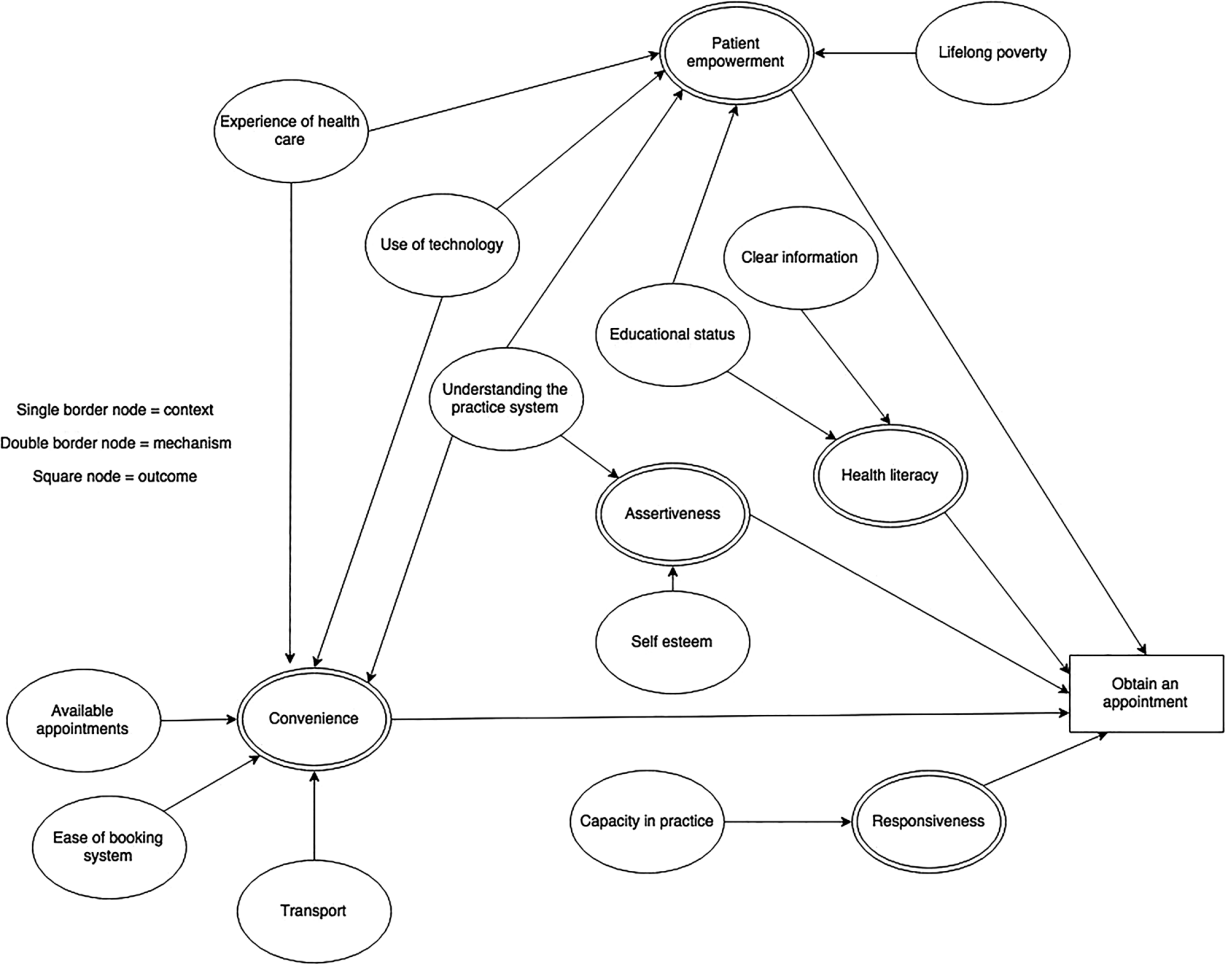
Context–mechanism–outcome configuration for obtain appointment.

**Figure 7:**
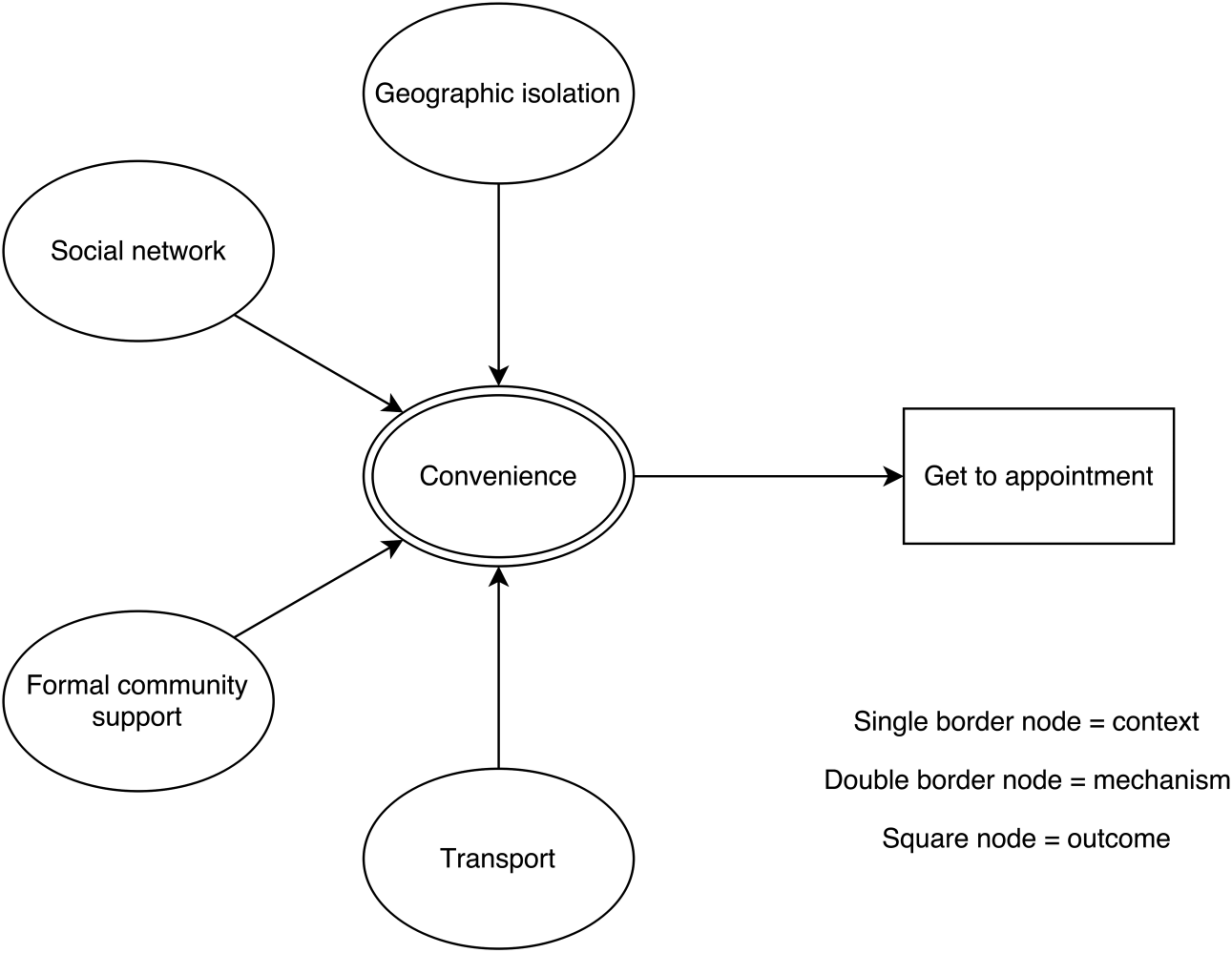
Context–mechanism–outcome configuration for get to appointment.

**Figure 8:**
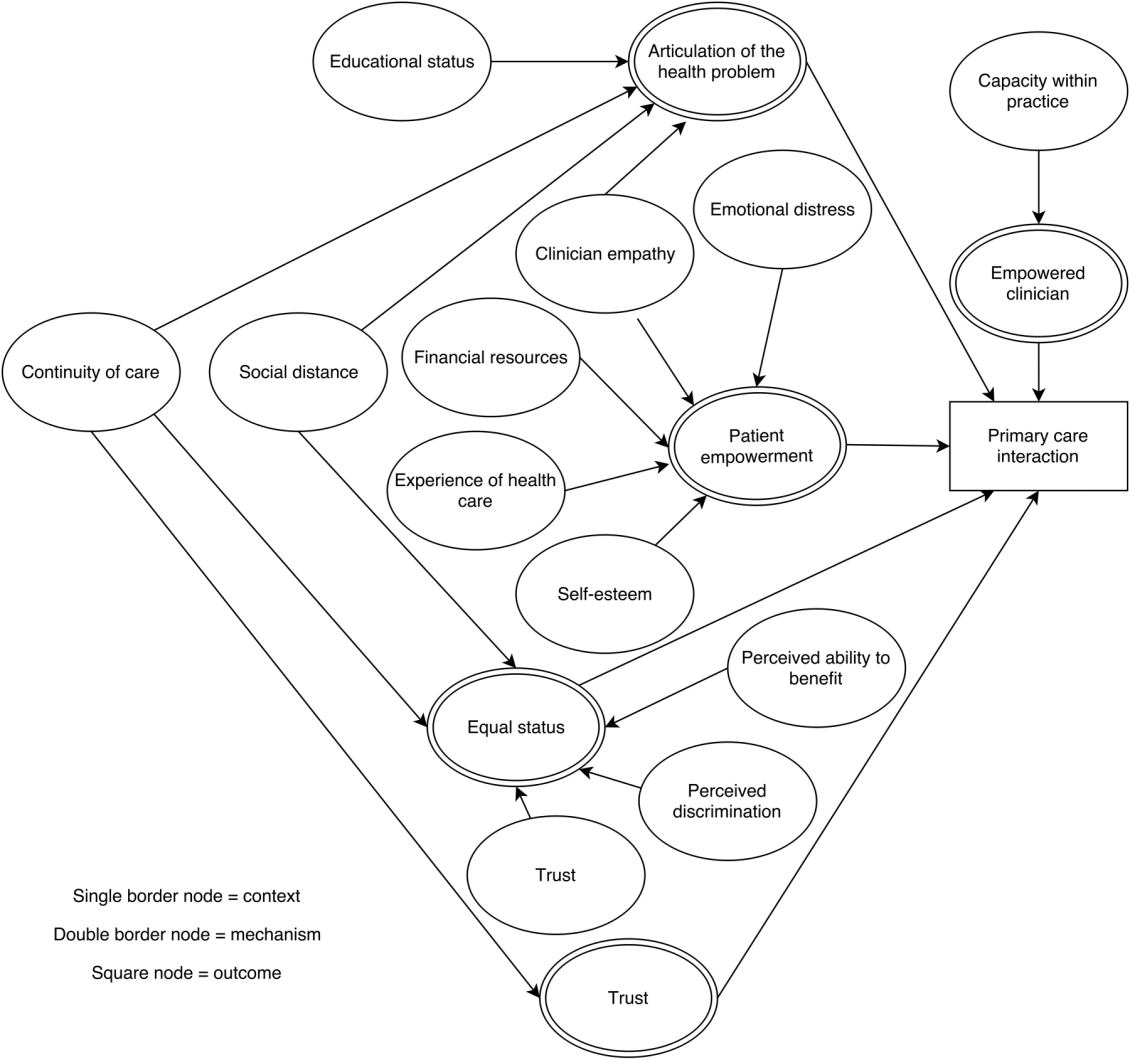
Context–mechanism–outcome configuration for primary care interaction.

For each of the steps in the patient pathway, we developed CMOCs which can be found unconfigured in tables 2–7 and configured in figures 3–8. Detailed explanation of how data from the literature contributed to each CMOC is shown in online supplementary appendix 2.

**Table 2:** Context–mechanism–outcome configuration for problem identified

Context	Mechanism	Outcome
Educational status^31^ ^32^ Health beliefs^33^ Problematic experience^34^ ^35^ Stoicism^33^ Social network^36^	Denial^33^ ^35^ Evaluation of evolving experiences^36^ Health literacy^31^ ^32^ ^34^	Problem identified

**Table 3:** Context–mechanism–outcome configuration for decision to seek help

Context	Mechanism	Outcome
Carer responsibilities^37^ ^38^ Expectations of ageing^39–42^ Experience of healthcare^39^ ^43–47^ Experience of symptoms^34^ ^39^ ^48^ Financial resources^37^ ^49^ ^50^ ^51^ Lifelong poverty^43^ ^52–55^ Perceived limited health resources^39^ Relevance of services^43^ ^56^ Self-esteem^40^ ^52^ ^57^ Social network^58^ ^59^ Stoicism^33^ ^35^ ^39^ ^60^ Transport^61^	Anxiety^34^ ^58^ Candidacy^39–41^ ^43^ ^44^ Convenience^37^ ^49^ ^61^ Denial^50^ ^51^ ^60^ Perceived ability to benefit^39^ ^40^ ^45^ Perceived ability to cope^35^ ^39^ Perceived control^42^ ^46^ ^53^ ^54^ ^57^ Perceived social exclusion^39^ ^47^ ^56^ ^55^ ^62^	Decision to seek help

**Table 4:** Context–mechanism–outcome configuration for actively seek help

Context	Mechanism	Outcome
Choice^32^ Clear information^32^ ^56^ ^63^ Educational status^64^ ^65^ Experience of healthcare^35^ ^37^ Extent to which practice is welcoming^33^ ^37^ ^66^ Relationship with GP^35^ ^67^ Self-efficacy^68^ Transport^69^	Affinity to a practice^33^ ^35^ ^37^ ^67^ Convenience^32^ ^69^ Health literacy^56^ ^64^ ^65^ Patient empowerment^33^ ^63^ ^68^ Perceived ability to benefit^32^ ^35^ ^66^	Actively seek help

GP, general practitioner.

**Table 5:** Context–mechanism–outcome configuration for obtain an appointment

Context	Mechanism	Outcome
Available appointments^70^ Capacity within practice^27^ Clear information^25^ Ease of booking system^33^ Educational status^65^ ^71^ Experience of health care^46^ Lifelong poverty^72^ Self-esteem^56^ Transport^73^ Understanding the practice system^33^ ^74^ Use of technology^75–77^	Assertiveness^33^ ^56^ Convenience^27^ ^33^ ^70^ ^73^ ^75^ ^76^ ^78^ Health literacy^25^ ^71^ Patient empowerment^46^ ^65^ ^72^ ^74^ ^77^ Responsiveness^27^	Obtain an appointment

**Table 6:** Context–mechanism–outcome configuration for get to appointment

Context	Mechanism	Outcome
Formal community support^61^ Geographic isolation^79^ ^80^ Social network^39^ Transport^69^ ^79^	Convenience^39^ ^61^ ^69^ ^79^ ^80^	Get to appointment

**Table 7:** Context–mechanism–outcome configuration for primary care interaction

Context	Mechanism	Outcome
Capacity within practice^81^ Clinician empathy^47^ ^67^ ^82^ Continuity of care^45^ Educational status^67^ Emotional distress^82^ Experience of healthcare^33^ Financial resources^83^ Perceived ability to benefit^71^ Perceived discrimination^39^ Self-esteem^56^ ^71^ ^83^ ^84^ Social distance^85^ ^86^ Trust in healthcare^47^ ^85^	Articulation of the health problem^45^ ^56^ ^67^ Empowered clinician^81^ Equal status^39^ ^47^ ^71^ ^62^ ^85^ Patient empowerment^33^ ^84^ ^83^ Trust^45^	Primary care interaction

The first step in the patient pathway is identification of a problem (table 2 and figure 3). Some socioeconomically disadvantaged older people in rural areas who are experiencing symptoms may not recognise them as a problem because of poor health literacy^31^ ^32^ ^34^ linked to lower educational status^31–33^ (eg, unaware that unintentional weight loss could be a sign of cancer) or low social interaction or denial^33^ ^35^ because of stoicism.^33^ Health literacy will also affect how an individual evaluates their experiences.^36^

After a problem has been identified, a patient will decide if they should seek help (table 3 and figure 4). For this group, important mechanisms appear to be candidacy,^39–41^ ^43^ ^44^ the effort required to attend an appointment,^37^ ^49^ ^61^ what the possible consequences will be,^34^ ^58^ if the service will meet their need^39^ ^40^ ^45^ and if they can continue to manage independently without needing to seek healthcare.^35^ ^39^ Contexts influencing these mechanisms include personal characteristics (such as educational status,^37^ expectations of ageing,^39–42^ stoicism^33^ ^35^ ^39^ ^50^ ^60^ and self-esteem);^40^ ^52^ ^57^ resources available (such as finances,^37^ ^49^ ^51^ support from friends and family,^33^ transport^61^ and carer responsibilities);^38^ perception of the health service (such as perceived limited resources within healthcare^39^) and experience of healthcare*.*^39^ ^44^ ^46^ ^47^

If a patient decides that a problem warrants healthcare, the next step is to actively seek help (table 4 and figure 5). A socioeconomically disadvantaged older person in a rural area is more likely to seek help from primary care if they feel a sense of belonging to a practice^33^ ^35^ ^37^ ^67^ which they are able to get to easily,^32^ ^56^ ^64^ ^65^ ^69^ believe it will be of help^32^ ^35^ ^66^ and are empowered.^33^ ^63^ ^68^ These mechanisms are influenced by experience of healthcare,^35^ ^67^ educational status,^64^ ^65^ personal resources such as self-efficacy^68^ and transport.^69^

Once the decision to seek primary care is made, a patient is required to obtain an appointment for most primary care services in the UK (table 5 and figure 6). Key contexts are available appointments,^70^ capacity within the practice,^27^ availability of clear information^25^ and ease of the booking system.^33^ A socioeconomically disadvantaged older person in a rural area is less likely to be able to obtain an appointment if they do not understand the system,^25^ ^71^ are not assertive,^33^ ^56^ appointments are not available at convenient times^33^ ^70^ ^73^ ^75^ ^87–89^ or the practice is not responsive to their needs.^27^ Other contributing contexts include available personal resources (such as transport,^73^ technology,^75–77^ educational status^65^ ^71^ and experience of healthcare.^46^ ^78^

After an appointment is booked, a patient needs to get there (table 6 and figure 7). Geographical isolation,^79^ ^80^ local support (either social^39^ or community^61^) and access to suitable transport^69^ ^79^ are all important in influencing decisions about convenience,^39^ ^61^ ^69^ ^79^ ^80^ and subsequent likelihood of attending the appointment for older people in this group.

The quality of the primary care interaction depends on patient and clinician factors (table 7 and figure 8). A socioeconomically disadvantaged older person in a rural area may face problems in articulating the health problem^45^ ^56^ ^67^ and feeling empowered^33^ ^83^ ^84^ to negotiate care. These were related to concepts such as continuity of care,^45^ educational status^67^ and experience of healthcare.^33^ The clinician needs to have empathy^67^ ^82^ and capacity within practice,^81^ to deliver the care that is required. Capacity includes having sufficient consultation time; evidence suggests that socioeconomically disadvantaged people experience shorter consultation times^90^ but may have difficulty in articulating health problems, increased anxiety or feel pressured by crowded waiting rooms.^85^ Both patient and clinician need equal status^39^ ^47^ ^71^ ^85^ ^86^ which is related to patient trust in the healthcare system,^47^ ^85^ consistency of care^47^ and social distance.^56^ ^71^ ^83^ ^84^ ^86^

## Discussion

### Statement of principal findings

Socioeconomically disadvantaged older people in rural areas face personal, community and healthcare barriers that limit their access to primary care. Key contexts identified in this review were stoicism, education status, expectations of ageing, financial resources, understanding of the system, access to suitable transport, capacity in primary care, the booking system and experience of healthcare. Key mechanisms underlying these contexts were health literacy, perceived convenience, patient empowerment and responsiveness of the practice. Realist review proved a useful approach for making sense of some of the complex and dynamic relationship of access because it allows exploration of the underlying mechanisms.

### Strengths and limitations

Strengths include a broad search strategy that was not limited to studies of socioeconomically disadvantaged older people in rural areas accessing primary care. This reduced the risk of missing major concepts which were not unique but were relevant to this patient group and meant that we could take a broad overview of the topic. Furthermore, the breadth allowed sense to be made of the behaviour of some of the mechanisms under the different contexts reported in the included articles. CMOC were discussed with patients to ensure there were no obvious gaps or inconsistencies. The nature of the programme theory developed means that it can be adapted to other populations to help health service design. Our review has demonstrated that, unlike many realist reviews and literature on realist methodologies which focus on a specific intervention or programme, realist reviews can be useful to aid the development of a programme theory—in this case one that explores drivers and barriers of access to healthcare.

The main limitation was the lack of evidence specifically focusing on socioeconomically disadvantaged older people in rural areas. To overcome this, we took a broad approach, and while this meant we did not miss important concepts, some issues may not be relevant to this group. Furthermore, a broad approach meant that we had more evidence to support the programme theory. Most of this was from cross-sectional studies which generally provided information on context and outcome, while qualitative studies provided data on mechanisms. Unsurprisingly, there were no randomised controlled trials because, while they were eligible, we were not looking at a specific intervention. We did not undertake any formal assessment of the methodological rigour of each manuscript included in the review. However, we did make global judgements about the trustworthiness of data within documents or studies we used to support our inferences. Overall, we judged data to be sufficiently trustworthy to enable refinement of our programme theory.

A further limitation was that the broad approach and nature of the data meant that each CMOC could not fully elucidate each complex interaction, nor could we differentiate which contexts or mechanisms were more important than others to achieve desired outcomes. While undertaking a realist review researchers would generally become more focused to contain the large volume of data emerging.^23^ We purposefully kept our review broad so as to include data on the whole patient pathway because we believed that a broader programme theory would be more useful in helping us to develop and test any future interventions. Since we were able to achieve sufficient conceptual saturation for the focus of this review, we did not undertake any additional searches. No significant alterations were made to our review processes as the review progressed. Furthermore, it was not always clear what the direction of effect was within the CMOs because the limited literature, and therefore we have presented neutral CMOs.

### Comparisons with existing literature

No other reviews exist in this population. Most previous work looking at access to healthcare (eg, Hoeck,^91^ Pong *et al*^29^) is based on the Aday and Andersen Framework,^22^ specifically their description of predisposing, enabling and need factors. There are similarities between our programme theory and the Aday and Andersen Framework. For example, most of our concepts could be categorised accordingly, such as educational status (predisposing), transport (enabling) and unmet need (need). However, by using realist methodology, we were able to explore underlying mechanisms and identify and understand which contexts need to be modified by interventions so as to increase the likelihood that desirable outcomes would occur. The Aday and Andersen Framework lacked this additional level of detail and understanding (and hence coherent rationale) to inform intervention design as it generally only includes contexts and outcomes. Uniquely, we have been able to develop a coherent and transferable explanation of the steps and causal processes (in the form of the realist programme theory) of access to healthcare using the specific population of socioeconomically disadvantaged older people in rural areas. This is important because we will use the findings from our review to design an intervention to address access issues faced by this population group of older people.

A comprehensive review of access to primary care looked quantitatively at whether barriers increased or decreased access for three areas: diabetes, episodic care and Pap testing.^15^ Our review has included similar concepts as this review, except for those relating to health insurance because we focused on relevance to the UK. However, we were more focused on understanding the underlying mechanism of, for example, the appointment system, rather than quantitatively describing each barrier. None of these studies mapped out access along a patient pathway from identifying a problem to primary care interaction. In contrast, we have developed a patient pathway which (1) allows a more targeted approach to address specific access problems and; (2) provides a coherent overview of access to primary care services.

### Recommendations

Some contexts identified in the review, such as educational status and lifelong poverty, require upstream policy interventions; however, contextual factors which may be amenable to health service interventions are detailed below. Not every person will necessarily benefit from all of the below contextual changes, but our findings suggest a focus on these potential barriers.

- Where there is a perception that the health system does not have sufficient resources, messages about the health services aimed at reducing unnecessary healthcare attendances and promoting self-management should be carefully phrased, so that they do not lead to vulnerable groups, who infrequently access primary care, feeling unwelcome or not entitled to health services. For example, a media campaign to encourage use of digital resources may inadvertently lead socioeconomically disadvantaged older people without IT skills feeling that health services are not relevant to them.
- Where patients have a negative experience of healthcare and are at risk of poor access, organisations need to ensure that these experiences are identified and addressed to help those patients remain engaged with the service.
- Where patients have carer responsibilities, opportunities for respite are needed to enable carers to attend appointments.
- Where there are areas with poor public transport, community transport schemes or satellite clinics are needed to help socioeconomically disadvantaged older people in rural areas get to their appointment, especially if they do not have a support network.
- Where there is a complex healthcare system, services should ensure that information is provided in plain English and in a format which is accessible to vulnerable people, especially regarding how to navigate the system.
- Where practices have overstretched booking systems, practices need to be responsive to the needs of vulnerable people, such as having a priority, one-stop telephone number or protected appointments at suitable times during the day, as socioeconomically disadvantaged older people in rural areas may not be assertive and are often stoical. A balance is needed between simple, clear information and processes for patients while being flexible and able to cater for different needs.
- Where there is limited capacity within practice, resources need to be prioritised to ensure that healthcare staff are able to spend the time needed to provide high-quality care to vulnerable groups which will improve their experience, keeping them engaged with primary healthcare.

## Conclusion

Our realist review of access to primary care for socioeconomically disadvantaged older people in rural areas identified key contexts such as stoicism, education status, expectations of ageing, financial resources, understanding the system, access to suitable transport, capacity within practice, the booking system and experience of healthcare. Important underlying mechanisms were health literacy, perceived convenience, patient empowerment and responsiveness of the practice. Some of these contextual influences on access to care act as barriers across the patient pathway but are amenable to change and interventions should aspire to address them.
