# Supplementary material for: Access to primary care for socioeconomically disadvantaged older people in rural areas: a realist review
Source: BMJ Open. 2016 May 17;6(5):e010652. doi: 10.1136/bmjopen-2015-010652 (PMC4874140; doi:10.1136/bmjopen-2015-010652)
Supplement: Supplementary data [file bmjopen-2015-010652supp.pdf]

Appendix 1: Search strategy used in MEDLINE

1. exp Primary Health care/
2. (primary adj health-care).tw.
3. (general practitioner* or general practice* or family practice* or family practitioner*).tw.
4. (primary adj2 care).tw.
5. (primary adj healthcare).tw.
6. exp Family Practice/
7. exp General Practitioners/
8. exp General Practice/
9. 1 or 2 or 3 or 4 or 5 or 6 or 7 or 8
10. exp Aged/
11. (old$ adj2 (people* or person or adult$)).tw.
12. elderly.tw.
13. exp frail elderly/
14. 10 or 11 or 12 or 13
15. ((health or social or socioeconomic$) adj2 (equalit$ or equit$ or determinant$ or disparit$ or inequality$ or inequit$)).tw.
16. (depriv$ or poverty or poor or socio-economically disadvantage*).tw.
17. exp Socioeconomic Factors/
18. exp vulnerable populations/
19. exp healthcare disparities/
20. 15 or 16 or 17 or 18 or 19
21. exp Health Services Accessibility/
22. (access or utili?sation).tw.
23. 21 or 22
24. 9 and 14 and 20 and 23

Appendix 2

N.B. Numerous articles contributed to each CMO configuration. Below the CMOCs are described and an illustrative example given of evidence that underpins the configuration. Contexts are shown in Italics and an illustrative example of the supporting literature in **bold**.

Problem identified

The CMO configuration for the first step in the programme theory (problem identified) is shown in Table 2 and Figure 3.

The mechanism of denial to identifying a problem was related with the contexts of stoicism, problematic experience and social network. **Coles 2010** describes results of focus groups with 82 middle aged and older men in a deprived area of the UK [1]. The authors describe how men's attitudes reflect the need to be masculine and stoic, denying pain, sickness and health care (*stoicism*). **Tod 2001** describes patients' experiences of heart disease in 14 semi-structured interviews [2]. The authors found that some patients limit or adapt their lifestyles in order to deny a health care need, such as reducing mild physical activity to avoid chest pain having to see a doctor (*problematic experience*). The Illness Action Model [3] highlights the importance of social interactions, such as someone noticing a problem which had been disregarded which in turn may be accepted, rejected or denied (*social network*).

The mechanism of health literacy (degree to which an individual can understand health information and services based on general or personal knowledge) was related with the contexts of problematic experience, social network and educational status. **Adamson 2003** describes a questionnaire study in which 1350 UK residents were given clinical vignettes and asked about health care utilisation [4]. The authors found that black respondents, those from lower socio-economic groups and women were more likely to report a health seeking behaviour when confronted with an unmet need (*problematic*

experience). Research suggests that lower socio-economic groups consult primary care more, but are referred less [5]. This could be because people with lower educational status perceive more problems, have lower thresholds or consult for more minor ailments (*educational status*). **Beckman 2013** presents a secondary analysis of routine data (over 800,000 population) from Sweden [6]. The authors found people from lower income and education groups had worse health outcomes and the authors argue that low health literacy is the likely explanation (*educational status*).

The Illness Action Model [3] contributed to our understanding of the contexts of stoicism, problematic experience, social network and the mechanisms of evaluation of evolving experiences and health literacy. Briefly the model describes the importance of being able to maintain social interactions as a competent partner during an illness or problematic experience. Experiences are iteratively evaluated based on a stock of general and personal knowledge, augmented with social interactions (*evaluation of evolving experiences, health literacy and social network*).

<u>Decision to seek help</u>

The CMO configuration for the second step in the programme theory (decision to seek help) is shown in Table 3 and Figure 4.

The mechanism of anxiety was linked with the contexts of experience of health care and social network. **Adamson 2003** presents a theoretical model about access to health care based on a questionnaire study (n=1350) [4]. The authors suggest that anxiety leads to seeking health care and is affected by past experience by influencing the interpretation of health care (*experience of health care*). This is supported by Tversky and Kahneman's theory of heuristics and bias for judging uncertainty which suggests that people make judgements based on perceived probability from previous experiences [7]. **Gardner 1999** presents findings from 16 semi-structured of patients with angina [8]. The authors found that fear of hospitals, operations and medical tests, based on the

experience of their peers, was related with barriers to access to health care (*social network*). **Horner 1994** presents findings from semi-structured interviews with 19 rural residents [9]. The authors found that advice from close friends and family contribute to the perceived seriousness of the condition (*social network*).

The mechanism of convenience was linked with contexts of transport, financial resources and carer responsibilities. **Goodridge 2011** presents findings from seven semi-structured interviews with rural residents with COPD [10]. The authors found that the requirement to travel longer distances for rural residents made some people reluctant to seek health care, even in urgent situations (*transport*). **Qu 2011** reported a survey of 479 primary care patients in America [11]. The authors found that a subgroup of patients who were older with less education and a lower income, had a high satisfaction of the convenience (*financial resources*). **Brabyn 2004** looked at access to GPs using Geographic Information Software in New Zealand [12]. The authors suggest that older people, if resources are available, move closer to their GP practice to shorten travel time and increase convenience (*financial resources*). A report for the National Co-ordinating Centre for NHS Service Delivery and Organisation by **Arksey 2003** undertook a literature review and consultation to assess the problems carers face in accessing health care [13]. Carers suggested they needed more flexible appointments because of their caring responsibilities *(carer responsibilities)*.

The mechanism of denial of the need to seek help was related with financial resources, stoicism and expectations of ageing. **Auchincloss 2001** looked at the National Health Interview Survey of Older People in America. The authors found that access problems increase with decreasing wealth and rurality [14]. The authors argue that a lack of life essentials (e.g. food and housing) would reduce a person's focus on their health needs because of other priorities and this in turn may lead to denial and reluctance to seek help (*financial resources*). **Johnson 1998** analysed 254 questionnaires of rural residents [15] according to the Andersen Framework [16]. The authors argue that the desire to be independent and resistant to outside help leads to a reluctance or denial to seek help until acutely

unwell (*stoicism*). **Dixon 2000** presents a discursive comparison of rural and urban health [17]. The authors argue that rural people tend to view health as absence of disease and only seek health care where they believe there is a cure (*expectations of ageing*).

The mechanism of perceived ability to benefit was related with expectations of ageing and experience of health care. **Bentley 2003** describes a mini-ethnographic study in the UK with nine key informants who were rural and elderly [18]. Certain problems were believed to be simply related with ageing which may lead to a decision not to seek health care (*expectations of ageing*). **Camillo 2004** presents an ethnographic study of older women [19]. The author found that many older women, based on previous experience, learnt that often their expectations of health care were not met (*experience of health care*).

The mechanism of perceived ability to cope was related with stoicism and social network. **Bentley 2003**, in a mini-ethnographic study, found that rural older people would not contact the doctor unless there was significant health problem because they wanted to manage themselves and not burden the NHS (*stoicism*) [18]. Similarly **Tod 2001**, in 14 semi-structured interviews with patients with angina, found that people valued strength, stoicism and the ability to be self-reliant (*stoicism*) [2]. The Network Episode Model [20] highlights the fact that personal social network provide information, advice, emotional support to interpret and access health care (*social network*).

The mechanism of perceived control was related with expectations of ageing, experience of health care, lifelong poverty and self-esteem. **Perrig-Chiello 1999** reported on control in older people from a cohort study of 442 participants [21]. The authors found that chance or destiny had a dominant role in an elderly person’s perspective of health. The authors also found that age is correlated with an increase in external control (*expectations of ageing*). The concept of external control is supported by Rotter’s theory of locus of control [22]. In an editorial **Calnan 2003** argues that old age has been medicalised leading to increased dependency and passivity [23]. The biomedical model of health care reinforces these concepts every time an older person seeks help (*experience of health care*).

Bosma 1999a found in a cross sectional study of 2174 participants from the Netherlands that low control partly originates in adverse socioeconomic conditions during childhood (*lifelong poverty*) [24]. In a subsequent discussion paper **Bosma 1999b** argues that low control is socioeconomic conditions in adulthood contribute to control later in life (*lifelong poverty*) [25]. **Bryant 2001** analysed 22 semi structured interviews of healthy older people [26]. The authors found that an individual's locus of control was closely related with self-esteem (*self-esteem*).

The mechanism of perceived social exclusion was related with lifelong poverty, experience of health care and perceived limited health resources. **Moskowitz 2013** used survey data of 11,105 patients from California [27]. The authors found that socioeconomically disadvantaged people received poorer communication than affluent people. The authors argue that primary care does not make services accessible to socioeconomically disadvantaged patients (*experience of health care*). **McNiece 1999** undertook a secondary analysis of a national survey of almost 72,000 patients [28]. The authors found that socioeconomic differences identified in younger patients persisted into later life (*lifelong poverty*). **Mazza 2011** reported 18 focus groups of people with low socioeconomic status [29]. The authors found that some patients described experiences where GPs were more interested in acute care and less interested in discussing long term preventative care because the doctor did not think it was necessary (*experience of health care*). In the mini-ethnographic study, **Bentley 2003**, described how older rural people were reluctant to use health care and preferred to cope because of the cost to the NHS (*perceived limited health resources*) [18].

The mechanism of candidacy (the ways in which older people's eligibility for medical attention and intervention is jointly negotiated between themselves and health services) was related with perceived limited health resources, experience of health care, expectations of ageing, relevance of services, lifelong poverty, experience of symptoms, social network and self-esteem. **Bentley 2003**, qualitatively looking at rural older people, reported that many felt that the GPs were always busy and this resulted in some playing down symptoms in order to help the GP's workload (*perceived*

limited health resources and experience of health care) [18]. **Campbell 1999** in a questionnaire survey of 4999 patients found that patients who thought there was poor GP availability had lower perception of what constituted urgent (*perceived limited health resources*) [30]. **Dixon-wood 2005** in a report to the National Co-ordinating Centre for NHS Service Delivery and Organisation (NCCSDO) describes the concept of candidacy [31]. The authors describe how perceived eligibility is constantly being defined and redefined through experiences (*experience of health care*). **Shipman 2009** reported interviews with older people with COPD, finding that older people often felt there was nothing the GP could do and this resulted in the individual postponing health seeking behaviour (*expectations of ageing*) [32]. **Ebrahim 1996** discussed issued facing marginalised older people [33]. He suggests that the cumulative effect over a lifetime of low incomes and a sense of isolation leads to older people perceiving services as irrelevant and insensitive to their needs (*relevance, lifelong poverty and experience of health care*). [31] **Bentley 2003** in a mini-ethnographic study of rural older people, found that when a symptom and past experience had resulted in, for example, antibiotics, the individual felt more confident to see the doctor again (*previous experience of symptoms*) [18]. The study also found that older people attribute symptoms to a normal part of ageing and do not consider them legitimate reasons to seek health care (*expectations of ageing*). **Coles 2010** in a qualitative study of 82 older deprived men found that men may legitimise seeking help through a female family member, overcoming the need to 'save face' (*social network*) [1]. **Jinks 2010** undertook semi-structure interviews with 28 older people with knee pain [34]. The authors found that an individual's upbringing or educational achievement effected how confident an individual was to seek health care (*lifelong poverty*). [30]**Shipman 2009** in a qualitative study of older people with COPD suggested a cycle where a worsening condition, led to loss of self-esteem and perception that their need for help is not valid (*self-esteem*) [32].

This CMO configuration was supported by the Network Episode Model which suggests that health seeking behaviour is mediated through social interactions and networks [20]. This could either be

through individual social interactions, such as through a personal social network, or at a macro level such as ones ongoing experience of healthcare.

Actively seek help

The CMO configuration for the third step in the programme theory (actively seek help) is shown in Table 4 and Figure 5.

The mechanism of affinity to a practice was related with relationship with GP and extent to which practice is welcoming. **Lamb 2012** used meta-synthesis to look at access problems for hard to reach groups [35]. The authors found that an understanding by the GP of who the patient was and how they related to the worlds they inhabit, based on previous interactions, was fundamental to their relationship with health care (*relationship with GP*). Similarly **Tod 2001** found that if the GP did not have a presence in the community, such as a single handed GP with several surgeries, then patients were more likely to delay seeking help (*relationship with GP*) [2]. **Coles 2010** found that some older men, based on the feel and atmosphere of the practice, felt that they were not welcome at some services (*extent to which practice is welcoming*) [1]. **Qu 2011** undertook a survey to look at the perception of staff in a practice who were not doctors (e.g. receptionists) [11]. In this study 40% of patients (n=479) expressed dissatisfaction about how these staff members facilitated access to doctors (*extent to which practice is welcoming and relationship with GP*).

The mechanism of convenience was related with clear information and transport. **Beckman 2013** in a study of routinely collected Swedish data (n=828,988) looked at access to primary care [6]. The authors argue that having clear information and knowledge of the alternatives are key to attaining the right solution for patients (*clear information*). Several studies described the impact of poor transport on access. One example was **Comber 2011** which combined an attitudes survey with GIS analysis and found that for those who did not own a car, the relative odds of experiencing difficulty in access to GPs was 3.8 times more than those ho did own a car (*transport*) [36].

The mechanism of health literacy was related with educational status and clear information. **Birch 1993** used routinely collected data to evaluate access to primary care in Canada [37]. The authors found that low levels of education were related with lower levels of use particular among patients with lower levels of need (*educational status*). Similarly **Bossuyt 2011** in a retrospective cohort study argue that a patient's educational attainment, via the mechanism of health literacy, is related with how health is sought (*educational status*) [38]. **Moskowitz 2013** looked at survey data from 11,105 Americans [27]. The authors found that doctors gave less information to lower socio-economic groups and this in turn is likely to influence how these patients are able to understand and navigate the health care system (*clear information*).

The mechanism of patient empowerment was related with extent to which the practice is welcoming, self-efficacy and clear information. **Coles 2010** found, based on focus groups with 82 socio-economically deprived older men, that increasingly positive experiences with health services led to an increase in assertiveness and empowerment (*extent to which practice is welcoming*) [1]. **Raymond 2011** undertook a cross sectional analysis of a randomised controlled trial of older people [39]. The authors argue that self-efficacy (an individual's optimistic self-belief) is related with a patient's ability to solve problems and is significantly less likely among women, those with basic education and those living alone (*self-efficacy*). **Freij 2011** undertook 25 qualitative interviews and six focus groups in older adults from America [40]. The authors found that when care co-ordinators gave clear information about available services patients were more confident to use services (*clear information*).

The mechanism of perceived ability to benefit was related with choice, the extent to which the practice is welcoming and experience of health care. **Beckman 2013**, in a Swedish cohort study, argue that choice is important to improving access because patients will be able to choose the best service for them based on availability, geographical location, opening hours, etc [6]. The authors also suggest that the ability to exercise choice is affected by income and/or education (*choice*).

Underwood 1994 reviewed interview transcripts of 46 deprived older people with experiences of cancer [41]. The authors found that some women were made to feel that their concerns about cancer and attendance were unwarranted. This type of experience is likely to make an individual feel unwelcome and of the opinion that primary care cannot meet their needs (*extent to which practice is welcoming*). **Tod 2001** in a qualitative interview of older people with angina found that negative previous experiences of accessing care resulted in a reduced likelihood of accessing the same care again (*experience of health care*) [2].

<u>*Obtain an appointment*</u>

The CMO configuration for the obtain an appointment step in the programme theory is shown in table 5 and Figure 6.

The mechanism of assertiveness was related with understanding the practice system and self-esteem. **Coles 2010** found that older men from deprived areas became more assertive as they learned to deal with the booking system (*understanding the practice system*) [1]. **Moskowitz 2013** argue that perceived social position, influenced by upbringing and life events, affects the assertiveness (*self-esteem*) [27].

The mechanism of convenience was related with available appointments, experience of health care, ease of booking system, understanding the practice system, use of technology and transport. Several studies described the impact of available appointments. For example, **Bennett 2009** report an analysis of routine appointment data (n=43,349), finding that patients who request an appointment but were not able to see their GP or had to wait more than 2 weeks were less likely to keep their appointment (*available appointments*) [42]. **Buetow 2002** presents data on 39 semi-structured interviews of people with asthma and poor access to primary care [43]. The authors compared "patient-centred time" and "practice-centred time", arguing that primary care is often organised

around the preferences of the practice rather than patients. For example, the working day and calendar are divided into units of fixed value and practices impose systems of time management designed to meet their needs (*experience of health care*). **Coles 2010** in a qualitative study of older men found that appointment systems were frequently illogical and hard to understand, especially if quick access was required [1]. For example one man was told the next available appointment was in a fortnight, but if he phoned the next day at 8.30am he might be able to get one that day (*ease of booking system*). The study also found that men described having to "break into" the system to be able to successfully navigate it (*understanding the practice system*). **Choi 2011** presents data regarding use of technology from the US National Health Interview Survey (n=27,731) [44]. The authors found that for older people of both genders the increased use of technology was related with increased access to GPs, specialists or allied health professionals (*use of technology*). **Thommasen 2006** in a retrospective cohort study (n=2,378) of patients accessing health services in British Columbia argued that doctors who work in rural areas are likely to rely more on technology, rather than face-to-face appointments, to maximise efficiency [45]. Therefore patients who are able to use technology will find the service more convenient to access (*use of technology*). **Cheung 2012** analysed 230,258 adults in the US National Health Interview Survey, comparing Medicaid (less affluent) with private insurance (more affluent) beneficiaries [46]. In considering barriers to primary care, the largest difference between these groups was transport and the authors argue that more convenient locations are needed to help Medicaid beneficiaries (*transport*). **Morgan 2011** describes an observational study of 639 GP practices in England [47]. The authors argue that satisfaction with a service is based more on convenience than capacity. Improving convenience is likely to improve satisfaction with services and subsequent access (*experience of health care*).

The mechanism of health literacy was related with clear information and educational status. **Kovandzic 2011** presents an analysis of 33 qualitative interviewers of how patients with mental health problems access primary care [48]. One of the two main barriers was a lack of effective information that is culturally sensitive with adequate content at the right time (*clear information*).

Rogowski 2008 analysed routinely collected data from older people in the US (n=20,227) [49]. The authors found that patients with low education were less able to navigate health care pathways (*educational status*).

The mechanism of patient empowerment was related with educational status, lifelong poverty, experience of health care, use of technology and understanding the practice system. **Bossuyt 2011** describes a retrospective cohort study of older people who accessed health care at the end of their life (n=2445) [38]. The authors found that less educated people had few transitions throughout the health system and the authors suggest this is because they were unable to organise desired care (*educational status*). **Drummond 2000** describes an analysis of attendees at an out of hours service in Glasgow (n=3193) [50]. The authors argue that patients who are more affluent have developed better negotiating skills over their lifetime and are therefore able to better overcome barriers (*lifelong poverty*). **Calnan 2003** in an editorial argues that older people's experience of health care is medicalised and predominantly biomedical [23]. Therefore ageing is portrayed as a medical problem, re-enforcing dependency and passivity (*experience of health care*). **Goodall 2010** describes findings of eight focus groups with older people living in South Australia [51]. The authors argue that information technology allows patients to engage in a meaningful and empowered manner both in terms of navigating the system and acquiring knowledge (*use of technology*). **Roos 1997** presents an analysis of the socio-economic characteristics and health status in a Canadian study of approximately 600,000 people [52]. The authors suggest that higher socio-economic status results in knowing treatment options and pathways and then being able to better negotiate and ask for a referral when necessary (*understanding the practice system*).

The mechanism of responsiveness was related with capacity within practice. **Buetow 2002** in 29 semi-structured interviews with patients with asthma found that practices were organised around their own capacity needs, rather than patients' needs [43]. The authors describes how practices need to be flexible to address barriers, such as opening hours, traditional appointment systems,

intolerance of missed appointments, long waiting times and inadequate consultation lengths (*capacity within practice*).

Getting to the appointment

The CMO configuration for getting to the appointment step is shown in Table 6 and Figure 7.

The mechanism of convenience was related with geographic isolation, transport, social network and formal community support. **Jatrana 2009** analysed data from Survey of Family, Income and Employment in New Zealand (n=18,320) and argued that while financial barriers were important to patients, isolation and lack of transportation were especially important to deprived groups (*geographical isolation and transport*) [53]. Furthermore **Turnbull 2008** in a geographic analysis of routinely collected data of out of hours telephone calls compared geographical location, deprivation and health care use (n=34,229) [54]. The authors found that in rural areas, deprived populations were least likely to receive the health care they needed (*geographical isolation*). Transport was a recurrent theme which several studies discussed. For example, **Comber 2011** in a GIS analysis of 8530 patients in Leicestershire found that patients who did not own a car, compared to those who did, were 3.8 times more likely to experience difficulties over accessing GP (*transport*) [36]. **Bentley 2003** in interviews with older people from rural areas found that the closure of local amenities and public transport led to older people finding it difficult to get to the surgery (*formal community support*) [18]. Furthermore **Goodridge 2011** described semi-structured interviews with older people living in rural areas with chronic respiratory illness [10]. The authors found that patients who did not have a car or access to a local volunteer driver had to wait and depend on the good will of friends of family to get to an appointment *(social network*).

Primary care interaction

The CMO configuration for the primary care interaction step is shown in Table 7 and Figure 8.

The mechanism of articulation of the health problem was related with educational status, clinician empathy, social distance and continuity of care. **Lamb 2012** presents a meta-synthesis looking at how vulnerable people access health care [35]. The authors found that vulnerable groups find it hard to articulate their problems. This was due to vulnerable groups being unable to communicate using professional models of illness (*educational status*) and doctors being unable to understand where patients are coming from (*clinician empathy*). **Moskowitz 2013** describes an analysis of survey data from the Diabetes Study of Northern California (n=11,105) [27]. The authors found that patients' sense of where they fall in the social hierarchy affected their communication (*social distance*). **Camillo 2004** presents a qualitative, ethnographic study of older women who experienced problems with access to health care [19]. The author found that continuity instilled a strong sense of trust and helped to facilitate better communication (*continuity of care*).

The mechanism of empowered clinician was related with capacity within practice. **Magan 2011** undertook a cross sectional analysis of routinely collected hospital data in 34 health districts in Spain [55]. The authors found that conditions sensitive to primary care intervention were positively correlated with a GP's workload suggesting that as workload increases a GP's ability to intervene decreases (*capacity within practice*).

The mechanism of equal status was related with continuity of care, trust in health care, perceived ability to benefit, social distance and perceived discrimination. **Mazza 2011** presents a qualitative study of 18 focus groups comparing high and low socio-economic status [29]. The authors found that if patients consulted with a number of different GPs it led to conflicting opinions and this resulted in scepticism and uncertainty (*continuity of care and trust in health care*). **Rogowski 2008** in an analysis of routinely collected data of older people in the USA (n=20,227) argued that lower socio-economic groups develop learned beliefs about health care [49]. These might include less confidence in the efficacy of the health care system (perceived ability to benefit). **Cawston 2007** describes

participatory action research in a deprived communicate in Scotland [56]. The authors found that a lack of respect, prejudice or labelling patients based on social group features led to social distance between the doctor and patient (*social distance*). **Bentley 2003** in interviews with older rural people found that some older people did not feel respected in regards to health care and that younger people got better treatment (*perceived discrimination*) [18].

The mechanism of patient empowerment was related with self-esteem, experience of health care, financial resources, clinician empathy and emotional distress. **Dixon 2007** presents a review of literature relating to equity in the NHS [57]. The authors argue that higher socio-economic groups have a louder “voice” because of better education and general self-confidence leading to a greater ability to persuade GPs to meet their needs (*self-esteem*). **Coles 2010** in focus groups with 82 older deprived men found that their confidence and trust grew with an increasing number of positive experiences (*experience of health care*) [1]. **Moffat 2004** presents findings from 11 semi-structured interviews of people receiving welfare advice [58]. The authors found that financial resources increased choice and control resulting in higher self-esteem and empowerment (*financial resources and self-esteem*). Mercer 2012 reports a questionnaire study of 3,044 patients attending 26 GPs in the UK [59]. The authors found that emotional distress and low GP empathy were associated with lower patient empowerment (clinician empathy and emotional distress).

The mechanism for trust was related with continuity of care. **Camillo 2004** presents a qualitative, ethnographic study of older women who experienced problems with access to health care [19]. The author found that continuity instilled a strong sense of trust and helped to facilitate better communication (*continuity of care*).

The primary care step was supported by Allport’s Contact Theory where contact is seen as important to promote understanding and reduce prejudice between groups [60]. This is important because socio-economically disadvantaged patients with low contact with their doctor will have fewer opportunities to reduce prejudice and social distance between themselves and their doctor.

References for appendix 2

1. Coles R, Watkins F, Swami V, et al. What men really want: a qualitative investigation of men's health needs from the Halton and St Helens Primary Care Trust men's health promotion project. Br J Health Psychol. 2010;15(Pt 4):921-39.
2. Tod AM, Read C, Lacey A, et al. Barriers to uptake of services for coronary heart disease: qualitative study. Bmj. 2001;323(7306):214.
3. Dingwall R, Robertson M. Aspects of Illness. Behavioural and Cognitive Psychotherapy. 1978;6(01):22.
4. Adamson J, Ben-Shlomo Y, Chaturvedi N, et al. Ethnicity, socio-economic position and gender--do they affect reported health-care seeking behaviour? Soc Sci Med. 2003;57(5):895-904.
5. Allin S, Hurley J. Inequity in publicly funded physician care: What is the role of private prescription drug insurance? Health Econ. 2009;18(10):1218-32.
6. Beckman A, Anell A. Changes in health care utilisation following a reform involving choice and privatisation in Swedish primary care: a five-year follow-up of GP-visits. BMC Health Services Research. 2013;13:452.
7. Tversky A, Kahneman D. Judgment under Uncertainty: Heuristics and Biases. Science (New York, NY). 1974;185(4157):1124-31.
8. Gardner K, Chapple A. Barriers to referral in patients with angina: qualitative study. Bmj. 1999;319(7207):418-21.
9. Horner SD, Ambrogne J, Coleman MA, et al. Traveling for Care: Factors Influencing Health Care Access for Rural Dwellers. Public Health Nurs. 1994;11(3):145-9.
10. Goodridge D, Hutchinson S, Wilson D, et al. Living in a rural area with advanced chronic respiratory illness: a qualitative study. Prim. 2011;20(1):54-8..
11. Qu H, Platonova EA, Kennedy KN, et al. Primary care patient satisfaction segmentation. Int J Health Care Qual Assur. 2011;24(7):564-76.
12. Brabyn L, Barnett R. Population need and geographical access to general practitioners in rural New Zealand. New Zealand Medical Journal. 2004;117(1199):U996.
13. Arksey H, Jackson K, Wallace AS, et al. Access to Health Care for Carers: Barriers and Interventions. London 2003.
14. Auchincloss AH, Van Nostrand JF, Ronsaville D. Access to health care for older persons in the United States: Personal, structural, and neighborhood characteristics. Journal of Aging and Health. 2001;13(3):329-54.
15. Johnson JE, Weinert C, Richardson JK. Rural residents' use of cardiac rehabilitation programs. Public Health Nurs. 1998;15(4):288-96.
16. Aday LA, Andersen R. A Framework for the Study of Access to Medical Care. Health Serv Res. 1974;9(3):208-20.
17. Dixon J, Welch N. Researching the rural-metropolitan health differential using the 'social determinants of health'. Aust J Rural Health. 2000;8(5):254-60.
18. Shipman C, White S, Gysels M, et al. Access to care in advanced COPD: factors that influence contact with general practice services. Prim. 2009;18(4):273-8.
19. Bentley JM. Barriers to accessing health care: the perspective of elderly people within a village community. Int J Nurs Stud. 2003;40(1):9-21.
20. Camillo P. Understanding the culture of primary health care: implications for clinical practice. J N Y State Nurses Assoc. 2004;35(1):14-9.
21. Pescosolido BA. Advances in Medical Sociology. JAI Press; 1995.

22. Perrig-Chiello P, Perrig WG, Stähelin HB. Health control beliefs in old age—relationship with subjective and objective health, and health behaviour. Psychology, Health & Medicine. 1999;4(1):83-94.
23. Rotter JB. Generalized expectancies for internal versus external control of reinforcement. Psychological monographs. 1966;80(1):1-28.
24. Calnan M. Are older people still grateful? Age and Ageing. 2003;32(2):125-6..
25. Bosma H, van de Mheen HD, Mackenbach JP. Social class in childhood and general health in adulthood: questionnaire study of contribution of psychological attributes. BMJ 1999;318(7175):18-22.
26. Bosma H, Schrijvers C, Mackenbach JP. Socioeconomic inequalities in mortality and importance of perceived control: cohort study. BMJ 1999;319(7223):1469-70.
27. Bryant LL, Corbett KK, Kutner JS. In their own words: A model of healthy aging. Social Science and Medicine. 2001;53(7):927-41..
28. Moskowitz D, Lyles CR, Karter AJ, et al. Patient reported interpersonal processes of care and perceived social position: The Diabetes Study of Northern California (DISTANCE). Patient Education and Counseling. 2013;90(3):392-8..
29. McNiece R, Majeed A. Socioeconomic differences in general practice consultation rates in patients aged 65 and over: prospective cohort study. BMJ 1999;319(7201):26-8.
30. Mazza D, Shand LK, Warren N, et al. General practice and preventive health care: a view through the eyes of community members. Medical Journal of Australia. 2011;195(4):180-3.
31. Campbell JL. Patients' perceptions of medical urgency: does deprivation matter? Fam Pract. 1999;16(1):28-32.
32. Dixon-Wood M, Kirk D, Agarwal S, et al. Vulnerable groups and access to health care: a critical interpretive review. London2005.
33. Ebrahim S. Ethnic elders. BMJ. 1996;313(7057):610-3.
34. Jinks C, Ong BN, O'Neill T. "Well, it's nobody's responsibility but my own." A qualitative study to explore views about the determinants of health and prevention of knee pain in older adults. BMC Public Health. 2010;10:148.
35. Lamb J, Bower P, Rogers A, et al. Access to mental health in primary care: a qualitative meta-synthesis of evidence from the experience of people from 'hard to reach' groups. Health (Lond). 2012;16(1):76-104..
36. Comber AJ, Brunsdon C, Radburn R. A spatial analysis of variations in health access: linking geography, socio-economic status and access perceptions. Int J Health Geogr. 2011;10:44..
37. Birch S, Eyles J, Newbold KB. Equitable access to health care: methodological extensions to the analysis of physician utilization in Canada. Health Econ. 1993;2(2):87-101.
38. Bossuyt N, Van den Block L, Cohen J, et al. Is individual educational level related to end-of-life care use? Results from a nationwide retrospective cohort study in Belgium. J Palliat Med. 2011;14(10):1135-41.
39. Raymond M, Iliffe S, Kharicha K, et al. Health risk appraisal for older people 5: self-efficacy in patient-doctor interactions. Prim Health Care Res Dev. 2011;12(4):348-56.
40. Freij M, Weiss L, Gass J, et al. "Just like I'm saving money in the bank": client perspectives on care coordination services. J Gerontol Soc Work. 2011;54(7):731-48..
41. Underwood SM, Hoskins D, Cummins T, et al. Obstacles to cancer care: focus on the economically disadvantaged. Oncol Nurs Forum. 1994;21(1):47-52.
42. Bennett KJ, Baxley EG. The effect of a carve-out advanced access scheduling system on no-show rates. Fam Med. 2009;41(1):51-6.
43. Buetow S, Adair V, Coster G, et al. Reasons for poor understanding of when and how to access GP care for childhood asthma in Auckland, New Zealand. Fam Pract. 2002;19(4):319-25.
44. Choi S. Longitudinal changes in access to health care by immigrant status among older adults: the importance of health insurance as a mediator. Gerontologist. 2011;51(2):156-69.

45. Thommasen HV, Tatlock J, Elliott R, et al. Review of salaried physician visits in a rural remote community - Bella Coola Valley. Canadian journal of rural medicine : the official journal of the Society of Rural Physicians of Canada = Journal canadien de la medecine rurale : le journal officiel de la Societe de medecine rurale du Canada. 2006;11(1):23-31.
46. Cheung PT, Wiler JL, Lowe RA, et al. National study of barriers to timely primary care and emergency department utilization among Medicaid beneficiaries. Ann Emerg Med. 2012;60(1):4-10.e2.
47. Morgan CL, Beerstecher HJ. Satisfaction, demand, and opening hours in primary care: an observational study. British Journal of General Practice. 2011;61(589):e498-507.
48. Kovandzic M, Chew-Graham C, Reeve J, et al. Access to primary mental health care for hard-to-reach groups: from 'silent suffering' to 'making it work'. Soc Sci Med. 2011;72(5):763-72.
49. Rogowski J, Freedman VA, Wickstrom SL, et al. Socioeconomic disparities in medical provider visits among medicare managed care enrollees. Inquiry. 2008;45(1):112-29.
50. Drummond N, McConnachie A, O'Donnell CA, et al. Social variation in reasons for contacting general practice out-of-hours: implications for daytime service provision? British Journal of General Practice. 2000;50(455):460-4.
51. Goodall K, Ward P, Newman L. Use of information and communication technology to provide health information: what do older migrants know, and what do they need to know? Qual Prim Care. 2010;18(1):27-32.
52. Roos NP, Mustard CA. Variation in health and health care use by socioeconomic status in Winnipeg, Canada: does the system work well? Yes and no. Milbank Q. 1997;75(1):89-111.
53. Jatrana S, Crampton P. Primary health care in New Zealand: who has access? Health Policy. 2009;93(1):1-10.
54. Turnbull J, Martin D, Lattimer V, et al. Does distance matter? Geographical variation in GP out-of-hours service use: an observational study. British Journal of General Practice. 2008;58(552):471-7.
55. Magan P, Alberquilla A, Otero A, , et al. Hospitalizations for ambulatory care sensitive conditions and quality of primary care: their relation with socioeconomic and health care variables in the Madrid regional health service (Spain). Med Care. 2011;49(1):17-23.
56. Cawston PG, Mercer SW, Barbour RS. Involving deprived communities in improving the quality of primary care services: does participatory action research work? BMC Health Services Research. 2007;7:88.
57. Dixon A, Le Grand J, Henderson J, et al. Is the British National Health Service equitable? The evidence on socioeconomic differences in utilization. J Health Serv Res Policy. 2007;12(2):104-9.
58. Moffatt S, White M, Stacy R, et al. The impact of welfare advice in primary care: A qualitative study. Critical Public Health. 2004;14(3):295-309.
59. Mercer SW, Jani BD, Maxwell M, et al. Patient enablement requires physician empathy: a cross-sectional study of general practice consultations in areas of high and low socioeconomic deprivation in Scotland. BMC Family Practice. 2012;13(1):1-9.
60. Allport GW. The Nature of Prejudice. Reading, MA: 1954.
